# Ice in the Rectum in Retention of Urine

**Published:** 1871-09

**Authors:** 


Ice in the Rectum in Retention of Urine.—Dr. Casenave says (*Jour. de Med. et de Chir.*) that during twenty years the following simple expedient has never failed in giving relief in retention of urine. He introduces into the rectum a piece of ice of the form of an elongated oval and about the size of a chestnut, which he pushes up beyond the sphincters, and renews every two hours. Almost always in an hour and a half, or two hours at longest, urethral spasm ceases, a certain quantity of urine is passed, and the bladder is emptied without effort by the patient. If in rare and exceptional cases this does not take place, he introduces again pieces of ice into the rectum, and places broken ice from the anus up to the end of the penis, until the urine flows, which it infallibly does. When there is difficulty in making water, occasioned by prostatic hypertrophy, the good effects of the ice are rather longer coming on, but almost always are produced. In short, in these circumstances (strictures and prostatic hypertrophies) the sedative effects are so well marked, thanks to the effects of the ice, that the introduction of bougies and sounds into the bladder and urethra is always rendered easy to practiced surgeons, and hardly any pain is felt.

